# Folate intake and colorectal cancer risk according to genetic subtypes defined by targeted tumor sequencing

**DOI:** 10.1016/j.ajcnut.2024.07.012

**Published:** 2024-07-16

**Authors:** Elom K Aglago, Conghui Qu, Sophia Harlid, Amanda I Phipps, Robert S Steinfelder, Shuji Ogino, Claire E Thomas, Li Hsu, Amanda E Toland, Hermann Brenner, Sonja I Berndt, Daniel D Buchanan, Peter T Campbell, Yin Cao, Andrew T Chan, David A Drew, Jane C Figueiredo, Amy J French, Steven Gallinger, Peter Georgeson, Marios Giannakis, Ellen L Goode, Stephen B Gruber, Marc J Gunter, Tabitha A Harrison, Michael Hoffmeister, Wen-Yi Huang, Meredith AJ Hullar, Jeroen R Huyghe, Mark A Jenkins, Brigid M Lynch, Victor Moreno, Neil Murphy, Christina C Newton, Jonathan A Nowak, Mireia Obón-Santacana, Wei Sun, Tomotaka Ugai, Caroline Y Um, Syed H Zaidi, Konstantinos K Tsilidis, Bethany van Guelpen, Ulrike Peters

**Affiliations:** 1 Department of Epidemiology and Biostatistics, Imperial College London, School of Public Health, London, United Kingdom; 2 Public Health Sciences Division, Fred Hutchinson Cancer Center, Seattle, WA, United States; 3 Department of Radiation Sciences, Oncology Unit, Umeå University, Umeå, Sweden; 4 Department of Epidemiology, University of Washington, Seattle, WA, United States; 5 Program in MPE Molecular Pathological Epidemiology, Department of Pathology, Brigham and Women's Hospital, Harvard Medical School, Boston, MA, United States; 6 Department of Oncologic Pathology, Dana-Farber Cancer Institute, Boston, MA, United States; 7 Department of Epidemiology, Harvard T.H. Chan School of Public Health, Boston, MA, United States; 8 Broad Institute of MIT and Harvard, Cambridge, MA, United States; 9 Department of Biostatistics, University of Washington, Seattle, WA, United States; 10 Department of Cancer Biology and Genetics and Internal Medicine, Comprehensive Cancer Center, The Ohio State University, Columbus, OH, United States; 11 Division of Clinical Epidemiology and Aging Research, German Cancer Research Center (DKFZ), Heidelberg, Germany; 12 Division of Preventive Oncology, German Cancer Research Center (DKFZ) and National Center for Tumor Diseases (NCT), Heidelberg, Germany; 13 German Cancer Consortium (DKTK), German Cancer Research Center (DKFZ), Heidelberg, Germany; 14 Division of Cancer Epidemiology and Genetics, National Cancer Institute, National Institutes of Health, Bethesda, MD, United States; 15 Colorectal Oncogenomics Group, Department of Clinical Pathology, Melbourne Medical School, The University of Melbourne, Parkville, VIC, Australia; 16 University of Melbourne Centre for Cancer Research, The University of Melbourne, Parkville, VIC, Australia; 17 Genomic Medicine and Family Cancer Clinic, The Royal Melbourne Hospital, Parkville, VIC, Australia; 18 Department of Epidemiology and Population Health, Albert Einstein College of Medicine, Bronx, NY, United States; 19 Division of Public Health Sciences, Department of Surgery, Washington University School of Medicine, St Louis, MO, United States; 20 Alvin J. Siteman Cancer Center at Barnes-Jewish Hospital and Washington University School of Medicine, St. Louis, MO, United States; 21 Division of Gastroenterology, Department of Medicine, Washington University School of Medicine, St. Louis, MO, United States; 22 Division of Gastroenterology, Massachusetts General Hospital and Harvard Medical School, Boston, MA, United States; 23 Channing Division of Network Medicine, Brigham and Women's Hospital and Harvard Medical School, Boston, MA, United States; 24 Clinical and Translational Epidemiology Unit, Massachusetts General Hospital and Harvard Medical School, Boston, MA, United States; 25 Department of Immunology and Infectious Diseases, Harvard T.H. Chan School of Public Health, Harvard University, Boston, MA, United States; 26 Department of Medicine, Samuel Oschin Comprehensive Cancer Institute, Cedars-Sinai Medical Center, Los Angeles, CA, United States; 27 Department of Population and Public Health Sciences, Keck School of Medicine, University of Southern California, Los Angeles, CA, United States; 28 Division of Laboratory Genetics, Department of Laboratory Medicine and Pathology, Mayo Clinic, Rochester, MN, United States; 29 Lunenfeld Tanenbaum Research Institute, Mount Sinai Hospital, University of Toronto, Toronto, ON, Canada; 30 Department of Medical Oncology, Dana-Farber Cancer Institute, Boston, MA, United States; 31 Department of Medicine, Brigham and Women's Hospital, Harvard Medical School, Boston, MA, United States; 32 Department of Quantitative Health Sciences, Division of Epidemiology, Mayo Clinic, Rochester, MN, United States; 33 Department of Medical Oncology & Therapeutics Research and Center for Precision Medicine, City of Hope National Medical Center, Duarte, CA, United States; 34 Centre for Epidemiology and Biostatistics, Melbourne School of Population and Global Health, The University of Melbourne, Melbourne, VIC, Australia; 35 Cancer Epidemiology Division, Cancer Council Victoria, Melbourne, VIC, Australia; 36 Unit of Biomarkers and Susceptibility (UBS), Oncology Data Analytics Program (ODAP), Catalan Institute of Oncology (ICO), L’Hospitalet del Llobregat, Barcelona, Spain; 37 ONCOBELL Program, Bellvitge Biomedical Research Institute (IDIBELL), L’Hospitalet de Llobregat, Barcelona, Spain; 38 Consortium for Biomedical Research in Epidemiology and Public Health (CIBERESP), Madrid, Spain; 39 Department of Clinical Sciences, Faculty of Medicine and health Sciences and Universitat de Barcelona Institute of Complex Systems (UBICS), University of Barcelona (UB), L’Hospitalet de Llobregat, Barcelona, Spain; 40 Nutrition and Metabolism Branch, International Agency for Research on Cancer, World Health Organization, Lyon, France; 41 Department of Population Science, American Cancer Society, Atlanta, Georgia; 42 Ontario Institute for Cancer Research, Toronto, ON, Canada; 43 Department of Hygiene and Epidemiology, University of Ioannina School of Medicine, Greece; 44 Wallenberg Centre for Molecular Medicine, Umeå University, Umeå, Sweden

**Keywords:** colorectal cancer, folate, folic acid, molecular subtypes, somatic mutations, tumor

## Abstract

**Background:**

Folate is involved in multiple genetic, epigenetic, and metabolic processes, and inadequate folate intake has been associated with an increased risk of cancer.

**Objective:**

We examined whether folate intake is differentially associated with colorectal cancer (CRC) risk according to somatic mutations in genes linked to CRC using targeted sequencing.

**Design:**

Participants within 2 large CRC consortia with available information on dietary folate, supplemental folic acid, and total folate intake were included. Colorectal tumor samples from cases were sequenced for the presence of nonsilent mutations in 105 genes and 6 signaling pathways (*IGF2/PI3K*, *MMR*, *RTK/RAS*, *TGF-β*, *WNT*, and *TP53/ATM*). Multinomial logistic regression models were analyzed comparing mutated/nonmutated CRC cases to controls to compute multivariable-adjusted odds ratios (ORs) with 95% confidence interval (CI). Heterogeneity of associations of mutated compared with nonmutated CRC cases was tested in case-only analyses using logistic regression. Analyses were performed separately in hypermutated and nonhypermutated tumors, because they exhibit different clinical behaviors.

**Results:**

We included 4339 CRC cases (702 hypermutated tumors, 16.2%) and 11,767 controls. Total folate intake was inversely associated with CRC risk (OR = 0.93; 95% CI: 0.90, 0.96). Among hypermutated tumors, 12 genes (*AXIN2*, *B2M*, *BCOR*, *CHD1*, *DOCK3*, *FBLN2*, *MAP3K21*, *POLD1*, *RYR1*, *TET2*, *UTP20*, and *ZNF521*) showed nominal statistical significance (*P* < 0.05) for heterogeneity by mutation status, but none remained significant after multiple testing correction. Among these genetic subtypes, the associations between folate variables and CRC were mostly inverse or toward the null, except for tumors mutated for *DOCK3* (supplemental folic acid), *CHD1* (total folate), and *ZNF521* (dietary folate) that showed positive associations. We did not observe differential associations in analyses among nonhypermutated tumors, or according to the signaling pathways.

**Conclusions:**

Folate intake was not differentially associated with CRC risk according to mutations in the genes explored. The nominally significant differential mutation effects observed in a few genes warrants further investigation.

## Introduction

Colorectal cancer (CRC) is 1 of the most diagnosed cancers worldwide, with an estimated global incidence of >1.9 million in 2020 [1]. CRC is a multifactorial disease with multiple established or putative genetic and modifiable risk factors [2]. Over the past decades, a substantial body of evidence from experimental and epidemiologic studies has suggested a possible protective role for folate in CRC [3]. Chemically, folate and folic acid constitute a group of compounds that possess a pterin ring conjugated to an aminobenzoate, and at least 1 glutamate moiety [4]. As an essential nutrient, folate is almost exclusively provided by the diet or through supplementation mostly in the form of folic acid [5].

Observational epidemiologic studies support a possible benefit offered by dietary folate and supplemental folic acid in CRC occurrence [6]. The most recent meta-analysis, which incorporated findings from 24 cohort studies, showed that high-folate intake was associated with 17% lower risk of developing CRC [7]. Previous meta-analyses on supplemental folic acid and CRC risk reported conflicting associations [6,8]. Great strides have been made in our understanding of the role of folate in colorectal carcinogenesis, with the most plausible pathway being folate’s role in 1-carbon metabolism, DNA methylation and synthesis [9].

Unlike most CRC risk factors that do not interact directly with DNA, folate is essential for the expression of key genes, nucleotide pool balance, DNA repair and epigenetic machinery, where folate acts as a cofactor for the synthesis of purines and thymidylate [10]. Suboptimal folate intake hypothetically contributes to defective DNA repair, hence promoting an accrual of gene mutations, genome instability, and higher CRC risk [11]. As a methyl donor, folate has a central role in both global DNA methylation, contributing to chromosomal stability, but also to gene-specific promoter methylation [12], acting as a regulator of gene expression [13]. Despite folate’s role in DNA methylation and purported role in CRC development, it is unclear whether folate differentially impacts somatic genes involved in colorectal carcinogenesis and what the general implications of folate are in the genetic architecture of colorectal tumors.

Here, we sought to investigate the relationships between total folate intake, and separately dietary and supplemental folic acid, and the risk of developing CRC according to acquired somatic mutations in 105 CRC-associated genes and 6 signaling pathways. To investigate this hypothesis, we used pooled data from case-control and cohort studies within 2 international consortia with available tumor tissue samples.

## Methods

### Study participants

Our study population consisted of participants diagnosed with CRC (cases) and controls in the Colorectal Cancer Family Registry, and Genetics and Epidemiology of Colorectal Cancer Consortium (GECCO), with available folate data and colorectal tumor samples (for cases). The design of each study, selection of controls, ascertainment of CRC cases, and the methods of data pooling and standardization has been extensively described [14,15]. Included studies and number of participants within each study are summarized in Supplemental Table 1.

CRC cases were defined as individuals who were diagnosed with an incident tumor in the colon or rectum, as confirmed by onco-pathologic records or provincial or state cancer registries, and/or death certificates.

### Ethical approval

Each participating study was approved by relevant ethics committees or review boards pertaining to their institutions. All participants provided informed consent at recruitment.

### Data collection and harmonization

Data on socio-demographics and lifestyle were collected via in-person interviews or structured self-administered questionnaires at baseline in cohort studies. In case-control studies, socio-demographics and lifestyles were collected at enrollment of control participants and recalled at a point in time 1–2 y before diagnosis for cases. Self-reported or measured anthropometric variables such as height and weight were also collected, as were medical history and dietary assessment. A multistep, iterative data harmonization procedure was undertaken to match each study’s unique protocol and collection instruments. The harmonization process was conducted centrally at the GECCO coordinating hub at the Fred Hutchinson Cancer Center, as previously described [15,16]. In brief, common data elements (CDEs) for variables such as sex or age or similar variables (e.g., smoking, dietary intake variables) were defined a priori for data harmonization. Each defined CDE is then unfolded on the basis of similarity and compatibility/comparability across studies, hence allowing statistical analysis across a combined dataset. Data harmonization used a dynamic communicative and feedback approach with data contributors to map study questionnaires and data dictionaries to these CDEs. Common definitions, permissible values, and standardized coding were implemented in a single database *via* SAS (version 9.4, SAS Institute; RRID:SCR_008567) and T-SQL. Multiple quality-control checks were performed, and outlying values within and between the studies were truncated to the minimum or maximum value of an established range for each variable. As an example, maximum height was set to 200 cm, and any participant with reported height above this value was set to 200 cm. This approach was used to prevent outliers for becoming influential points in the analysis.

### Folate status assessment

Usual diet was assessed in each participating study using food-frequency questionnaires or diet history questionnaires. Folate intake was estimated within each study by linking food items consumption and portion sizes with nutrient databases, while accounting for the introduction of cereal grains fortification in folic acid, when applicable (e.g., in United States studies after the year 1998) [17]. This was the case for the Prostate, Lung, Colorectal, & Ovarian Cancer Screening Trial. Folate intake for each participant was determined on the basis of folate content in each food item consumed (folate from foods, in μg/d), and folic acid (yes or no) was determined from dietary supplements either from single or multiple supplements. We calculated total folate (as dietary folate equivalent, in μg/d) as the sum of dietary folate and supplemental folate (intake of supplemental folic acid multiplied by a factor of 1.7 to account for higher bioavailability of folic acid compared with dietary folate) [18]. To estimate supplemental folic acid, actual quantities contained in the supplements were applied when available; otherwise, standard folate content of commercially available folic acid that was assumed to be 400 μg/d was used. In United States-based studies (e.g., Women’s Health Initiative), in which the recruitment of the participants spanned across folic acid fortification years (1996–1998), folic acid from fortified foods was accounted for by including 1.7 times folic acid from fortified foods to the total sum. Before modeling, characteristics related to dietary folate and total folate intake (μg) were energy-adjusted (dividing by total energy intake) and categorized as sex- and study-specific quartiles.

### Targeted sequencing

DNA extraction and targeted genome sequencing for somatic alterations was conducted as previously described elsewhere [19]. In brief, tissue samples with <70% tumor content were macrodissected from slides guided by hematoxylin and eosin-staining marked for the tumor regions. DNA concentrations were determined by Quant-iT PicoGreen dsDNA Assay or the Qubit dsDNA HS Assay kits. DNA sequencing libraries were barcoded and then pooled in 48 or 192 samples for sequencing on a HiSeq 2500 (Illumina HiSeq 2500, Illumina). Low-yield samples were topped up with additional sequencing where needed. Using Burrows-Wheeler Aligner (BWA-MEM version 0.7.9), paired-end reads were aligned to the reference human genome (GRCh37/hg19). On the aligned data, local realignments and base quality recalibrations were performed. For downstream analysis, we utilized only reads that were uniquely matched to the reference human GRCh37/hg19 genome assembly. We identified somatic single nucleotide variations (SNVs) using Strelka v1.0.15 [20] and MuTect v1.1.7 [21] and used Annotate Variation (ANNOVAR) to annotate somatic mutation calls, including additional filters such as read-depth, alternative read-depth, clustered read location, strand bias, and minor allele frequency in Exome Aggregation Consortium. We plotted point mutations for all samples to determine their hypermutation status, and we noticed 2 distinct peaks. We defined hypermutation status by using the minimum value of 23-point mutations per sample (17 mutations per million bases) between the 2 peaks [19]. We obtained insertion/deletion (indel) calls using majority votes from VarScan2 v2.4.349, VarDict (Feb 2017), and Strelka v1.0.1547. After initial filtering of indels on the basis of coverage and mutant allele frequency, we noticed some background signals of alternative reads in normal samples. Thus, we used read counts from tumors and normal samples to construct a background filter to remove indel calls in a subset of samples where signals were not significantly higher than background. We evaluated calls for 91 indels and 96-point mutations chosen at random using Sanger sequencing [22]. Following that, we carried out a validation study employing Sequenom (Laboratory Corporation of America) as an orthogonal technology for indels and point mutations. For point mutation calls, we observed false positive and false negative rates of 0.3% and 4.1%, respectively, with a sensitivity of 95.9% and a specificity of 99.7%.

We used the data to fine-tune our calling algorithms further because the validation for indels revealed room for improvement [19]. For a second validation of 109 indels, subsequent Sanger sequencing revealed 93.6% correct calls and for SNVs, we tested 84 mutations by Sanger sequencing and showed 98.8% correct calls. On the basis of ANNOVAR refGene annotations [23], we defined gene mutations as the presence of nonsilent mutations. If an SNV was annotated as exonic and nonsynonymous, stop-gain, stop-loss, or splicing, it was considered nonsilent. If an indel was annotated as exonic and included a frameshift deletion, frameshift insertion, in-frame deletion, in-frame insertion, stop-gain, or stop-loss, it was considered nonsilent. A total of 227 genes and 6 common signaling pathways (*IGF2/PI3K*, *MMR*, *RTK/RAS*, *TGF-β*, *WNT*, and *TP53/ATM*) were tested.

### Statistical analyses

We computed odds ratios (ORs) with 95% confidence intervals (CIs) separately for dietary folate, supplemental folic acid, and total folate associated with CRC risk (reference category was “No” for supplemental folic acid, and 1 μg/1000 Kcal/d increment for dietary and total folate) using logistic regression in each study. We performed individual patient meta-analysis to calculate OR and 95% CI for folate variables and CRC in all participants.

**FIGURE 1:**
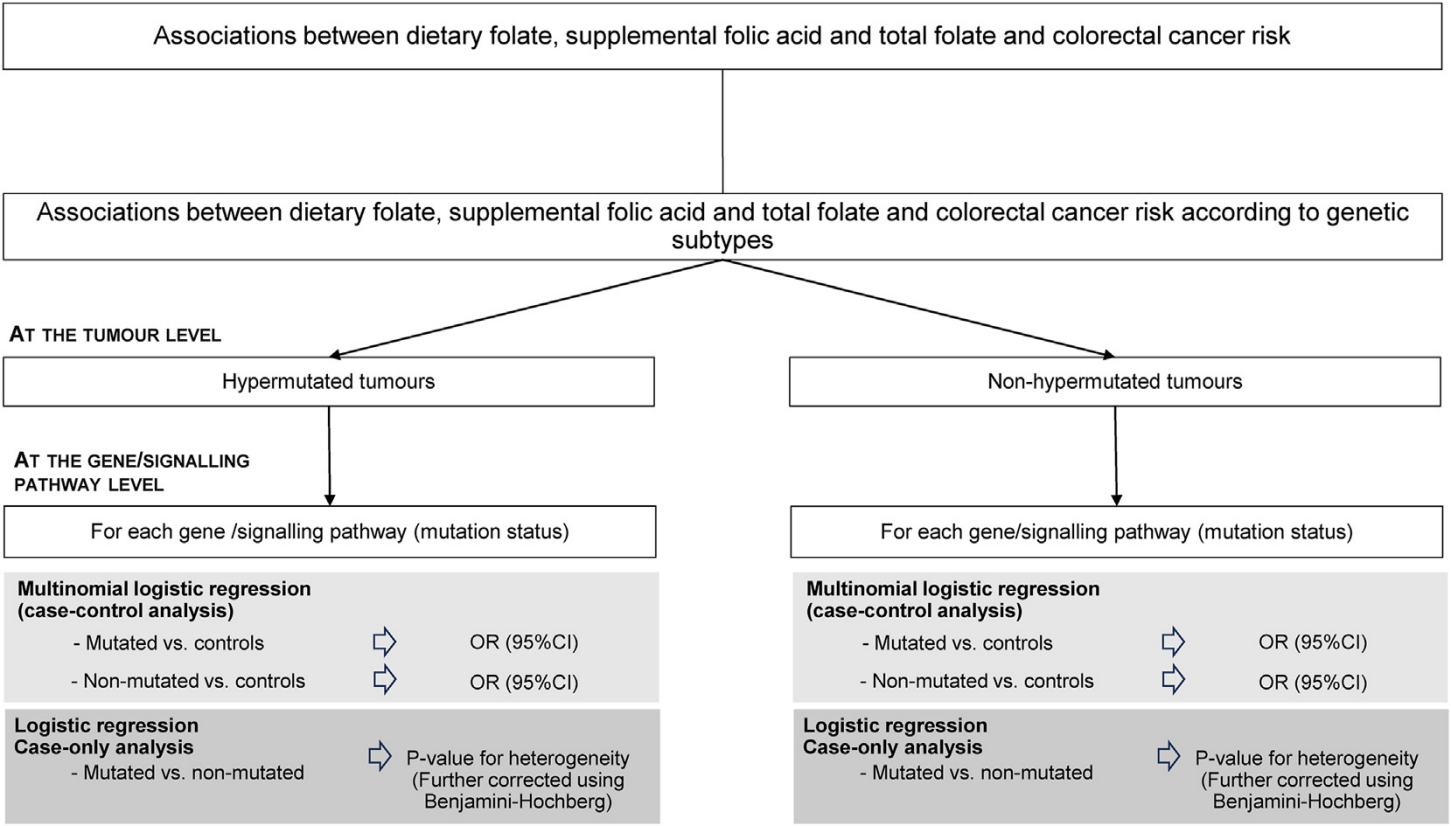
Summary of the statistical analysis for the associations of folate variables and CRC by somatic genetic subtypes. The approaches used for the statistical analyses for the associations between dietary folate, supplemental folic acid, and total folate by genetic subtypes was presented. CRC, colorectal cancer.

The statistical analysis for the associations of folate variables and CRC by genetic subtypes is summarized in Figure 1. We used multinomial logistic regression models and pooled individual-level data to compute ORs (95% CIs) for the association between dietary folate, supplemental folic acid, and total folate and the risk of CRC according to mutated and nonmutated gene status of cases compared with control participants. In case-only analyses, logistic regression models were analyzed to examine heterogeneity of folate variables associations between mutated compared with nonmutated tumors, using *P* value of the differential association as *P* for heterogeneity. Only these *P* values are presented from the case-only analyses, following a consistent pattern of presentation in other relevant GECCO publications [[24], [25], [26], [27]].We considered *P* values <0.05 as nominally significant. We conducted the analyses separately for hypermutated and nonhypermutated tumors. The rationale for the separate consideration of hypermutated and nonhypermutated tumors was motivated by the fact that hypermutated tumors exhibit a different behavior, because they are more likely to arise in right-sided colon, are less likely to be diagnosed at stage IV, and have more favorable CRC-specific survival than nonhypermutated tumors [19]. Among nonhypermutated tumors, the analyses were restricted to the genes mutated in ≥5% of total cases, whereas in hypermutated tumors, the analyses were conducted in genes mutated in ≥15% of the cases. Of the 227 mutated genes in hypermutated tumors, 93 genes reached the 15% threshold and were included in our analyses, whereas in nonhypermutated tumors, 12 genes reached the threshold of 5% of mutated cases and were included in our analyses. The rationale to restrict the analyses to this set of genes was to streamline the analysis to the most important genes with sufficient statistical power. Previous investigations in GECCO demonstrated that hypermutation is driven by mutations in more genes and displayed more alterations in multiple pathways, compared with nonhypermutation status, which is driven by fewer genes [19]. Therefore, for each gene, we calculated the percentage of mutated cases over nonmutated cases, separately in hypermutated and nonhypermutated tumors and retained only genes that passed the 15% and 5% threshold, respectively.

We adjusted our analyses for variables selected a priori including study (except in the meta-analyses of folate and overall CRC risk), age (in years, continuous), sex (female or male), smoking (yes or no), alcohol consumption (nondrinker, 1–28 g/d, ≤2 drinks/d; 28–42 g/d, >2–≤3 drinks/d; >42 g/d, or >3 drinks/d), physical activity (active, inactive, or missing), family history of CRC (yes, no, or missing), diabetes (yes, no, or missing), BMI (normal, overweight, obese, or missing), and dietary intakes of fiber (g/d; continuous), processed meat (servings/d; continuous), fruits (servings/d; continuous), and vegetables (servings/d; continuous). We further applied Benjamini–Hochberg false discovery rate control at 0.05 to account for multiple testing. All analyses were performed using R version 4.1.2 (R Project for Statistical Computing, RRID:SCR_001905)[28].

## Results

**FIGURE 2:**
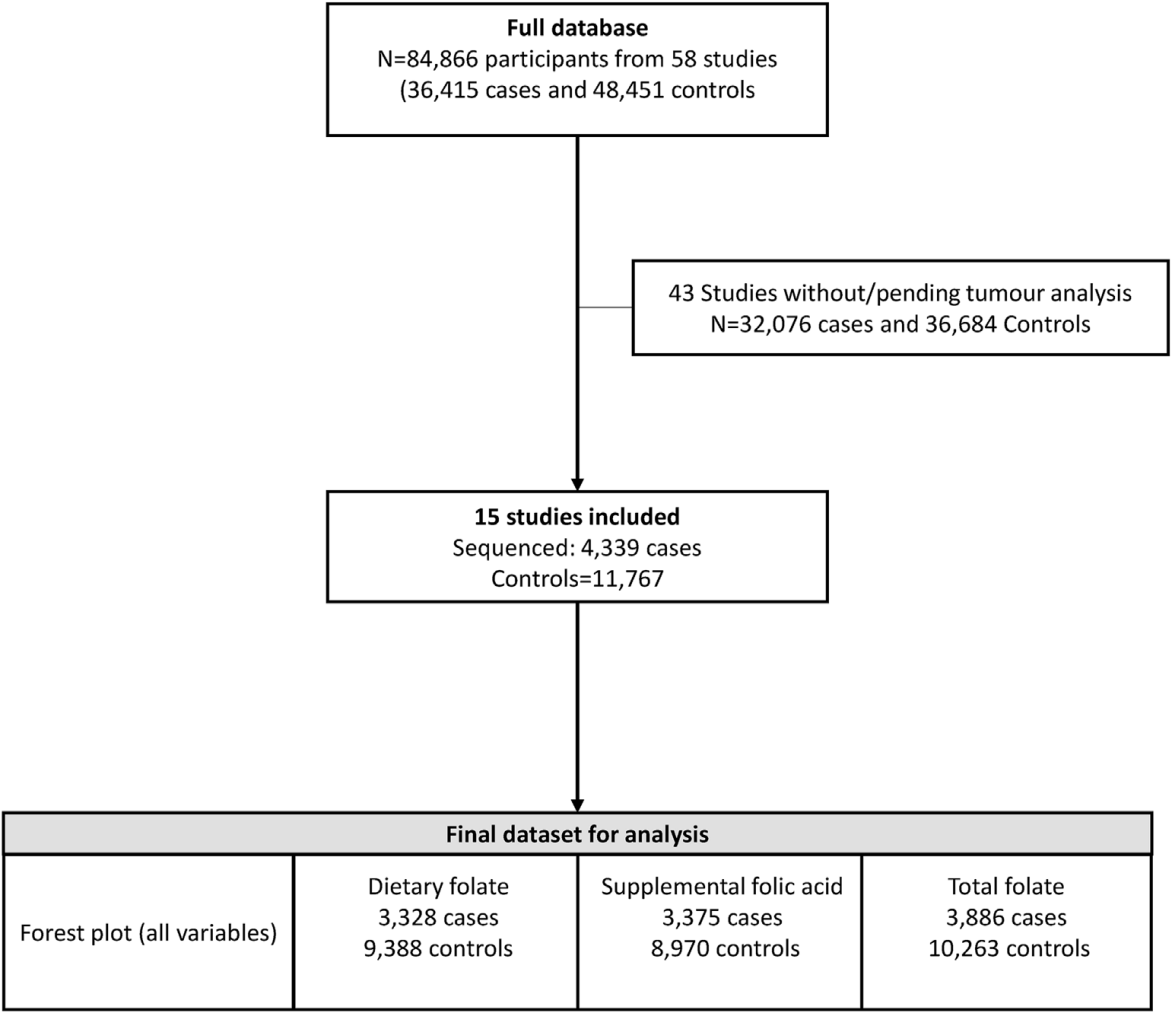
Flowchart of inclusion of the participants. Number of studies and participants included in the final analytical dataset.

**FIGURE 3:**
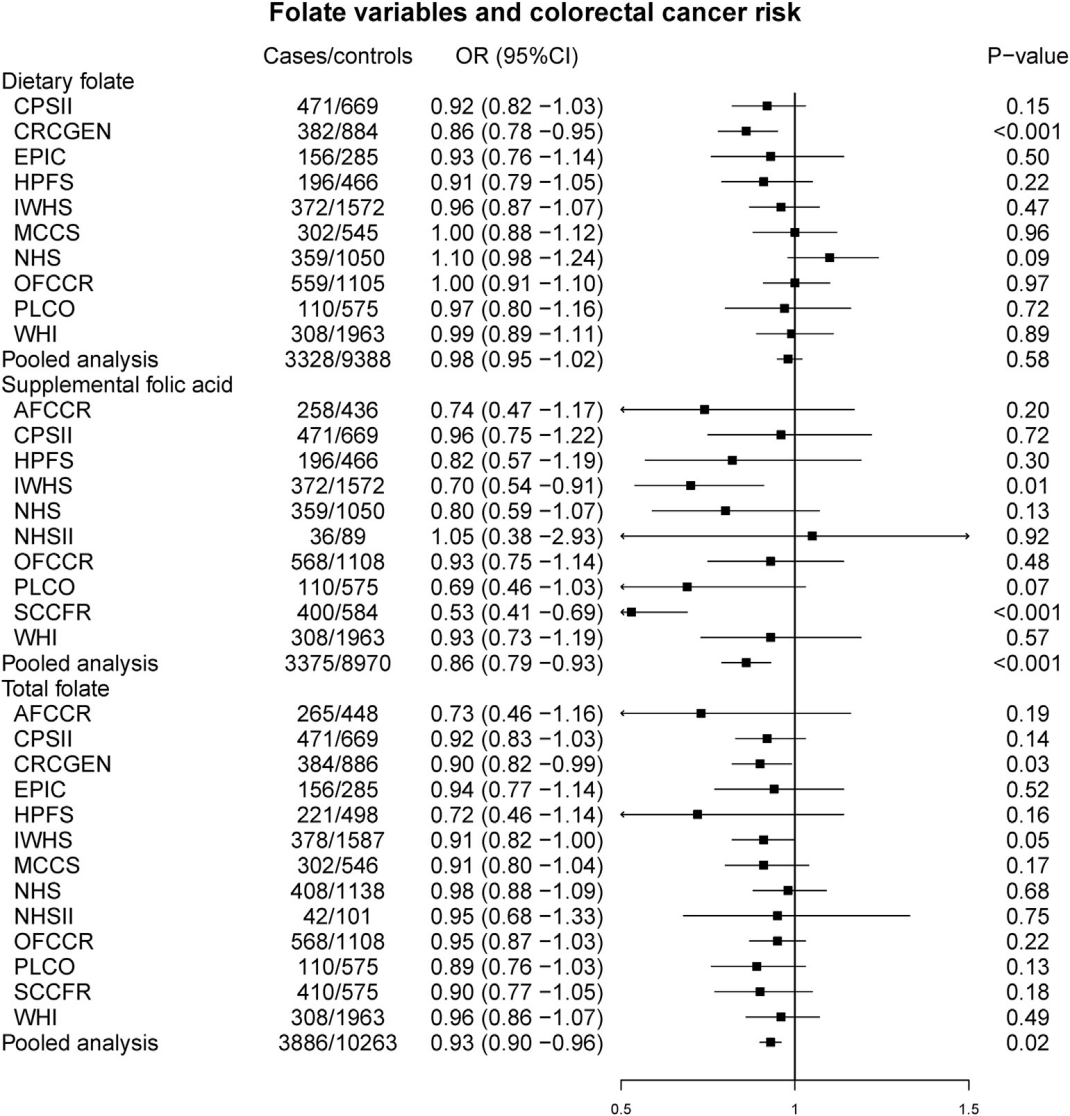
Forest plot for folate variables and colorectal cancer risk. Odds ratios and 95% confidence intervals for dietary folate, supplemental folic acid, and total folate associated with colorectal cancer for each participating study.

A total of 4339 CRC cases (702 had hypermutated tumors, 16.2%) and 11,767 controls were included in the final analysis (Table 1, Figure 2). Total folate intake was inversely associated with CRC risk (OR: 0.93; 95% CI: 0.90, 0.96) (Figure 3). The inverse association observed between total folate and CRC risk was driven by supplemental folic acid intake (OR: 0.86; 95% CI: 0.79, 0.93), and the association was null for dietary folate intake (OR: 0.98; 95% CI: 0.95, 1.02).

**TABLE 1:** Characteristics of the study case and control populations

	Cases, *N* = 4339	Controls, *N* = 11,767
Age, y	66.2 ± 12.0	67.3 ± 11.5
Sex, *n* (%)		
Female	2768 (63.8)	8153 (69.3)
Male	1571 (36.2)	3614 (30.7)
Family history of CRC, *n* (%)		
No	3338 (76.9)	9820 (83.5)
Yes	727 (16.8)	1226 (10.4)
Missing	274 (6.3)	721 (6.1)
Smoking, ever, *n* (%)		
No	2146 (49.5)	6354 (54)
Yes	2193 (50.5)	5413 (46)
Alcohol, *n* (%)		
Nondrinker	2200 (50.7)	5676 (48.2)
1–28 g/d (1 to ≤2 drinks/d)	1620 (37.3)	5108 (43.4)
>28 g/d (>2 drinks/d)	414 (9.5)	596 (5.1)
Missing	105 (2.4)	387 (3.3)
Physical activity, *n* (%)		
Inactive	419 (9.7)	1962 (16.7)
Active	784 (18.1)	2324 (19.8)
Missing	3136 (72.3)	7481 (63.6)
BMI, *n* (%)		
Normal	1534 (35.4)	4690 (39.9)
Overweight	1694 (39.0)	4553 (38.7)
Obese	987 (22.7)	2237 (19.0)
Missing	124 (2.9)	287 (2.4)
Diabetes, *n* (%)		
No	3827 (88.2)	10,878 (92.4)
Yes	427 (9.8)	837 (7.1)
Missing	85 (2)	52 (0.4)
Tumor location		
Colon	3224 (74.3)	NA
Rectum	1115 (25.7)	NA
Tumor stage, *n* (%)		
Stage I or local	919 (21.2)	NA
Stage II/III or regional	2556 (58.9)	NA
Stage IV or distant	409 (9.4)	NA
Missing	455 (10.5)	NA
Red meat, servings/d	0.80 ± 0.67	0.75 ± 0.6
Processed meat, servings/d	0.36 ± 0.39	0.35 ± 0.37
Vegetable, servings/d	2.81 ± 2.09	2.71 ± 1.96
Fruit, servings/d	2.02 ± 1.73	2.09 ± 1.62
Fiber, g/d	20.7 ± 11	18.6 ± 10.6
Dietary folate, μg/d	412 ± 238	419 ± 226
Total folate, μg/d	598 ± 573	678 ± 558
Supplemental folic acid, *n* (%)		
No	1804 (41.6)	4295 (36.5)
Yes	1440 (33.2)	4217 (35.8)
Missing	1095 (25.2)	3255 (27.7)

Abbreviations: BMI, body mass index; CRC, colorectal cancer

There are no missing values for a variable, unless otherwise specified in the table.

In hypermutated tumors, we did not observe any difference (heterogeneity) in the associations between folate intake and mutational status of the evaluated genes after accounting for multiple testing (All *Q*-values > 0.05) (Supplemental Tables 2–4). Nevertheless, when we considered a nominal association threshold (*P* < 0.05), we found heterogeneity with dietary folate and 3 genes [chromodomain-helicase-DNA-binding protein 1 (*CHD1*); tet methylcytosine dioxygenase 2 (*TET2*); and zinc finger protein 521 (*ZNF521*)], supplemental folic acid and 4 genes [beta-2-microglobulin (*B2M*); dedicator of cytokinesis 3 (*DOCK3*); Fibulin 2 (*FBLN2*); polymerase delta 1 (*POLD1*)], and total folate with 6 genes [axin 2 (*AXIN2*); BCL6 corepressor (*BCOR*); mitogen-activated protein kinase kinase kinase 21, *MAP3K21*; ryanodine receptor 1 (*RYR1*); small subunit processome component 20 (*UTP20*)*;* and *ZNF521*) (Table 2). The associations between folate variables and CRC risk were mostly inverse or toward the null, irrespective of the mutation status of the genes tested. However, for a few genes, folate variables were positively associated with mutated gene status, whereas inverse or null associations were found for the nonmutated genes. These were observed for 3 genes: *ZNF521* (total and dietary folate), *CHD1* (total folate), and *DOCK3* (supplemental folic acid). The highest OR was noted for supplemental folic acid intake in relation to *DOCK3*-mutated CRC risk (OR: 1.88; 95% CI: 1.09, 3.25).

**TABLE 2:** Summary associations between folate variables and colorectal cancer risk according to mutations in specific genes in hypermutated tumors

Outcome	Mutated cases vs. controls				Nonmutated cases vs. controls				*P* for heterogeneity^1^	BH corrected *P* value^2^
	Cases	Controls	OR (95% CI)	*P* value	Cases	Controls	OR (95% CI)	*P* value		
Dietary folate										
*ZNF521*	96	9388	1.38 (1.02, 1.87)	0.039	534	9388	0.95 (0.83, 1.09)	0.444	0.002	0.318
*TET2*	126	9388	0.72 (0.54, 0.96)	0.025	504	9388	1.08 (0.94, 1.24)	0.270	0.019	0.716
*CHD1*	73	9388	1.48 (1.07, 2.06)	0.019	283	9388	0.92 (0.77, 1.11)	0.397	0.037	0.876
Supplemental folic acid										
*DOCK3*	77	8970	1.88 (1.09, 3.25)	0.023	203	8970	0.63 (0.45, 0.88)	0.007	0.004	0.398
*B2M*	166	8970	1.20 (0.86, 1.68)	0.284	475	8970	0.67 (0.55, 0.82)	<0.001	0.020	0.716
*POLD1*	169	8970	0.59 (0.42, 0.82)	0.002	472	8970	0.86 (0.7, 1.05)	0.128	0.026	0.716
*FBLN2*	120	8970	0.55 (0.37, 0.83)	0.004	521	8970	0.84 (0.69, 1.01)	0.069	0.027	0.716
Total folate										
*ZNF521*	110	10,263	1.23 (0.93, 1.64)	0.143	592	10,263	0.82 (0.73, 0.92)	0.001	0.002	0.318
*MAP3K21*	42	10,263	0.45 (0.28, 0.73)	0.001	272	10,263	0.85 (0.71, 1.02)	0.075	0.005	0.398
*AXIN2*	178	10,263	0.69 (0.56, 0.85)	0.000	524	10,263	0.94 (0.83, 1.07)	0.348	0.008	0.509
*UTP20*	138	10,263	0.71 (0.55, 0.91)	0.008	564	10,263	0.91 (0.81, 1.02)	0.110	0.025	0.716
*BCOR*	178	10,263	0.73 (0.58, 0.9)	0.003	524	10,263	0.92 (0.82, 1.04)	0.192	0.025	0.716
*RYR1*	360	10,263	0.81 (0.7, 0.95)	0.008	342	10,263	0.92 (0.8, 1.07)	0.284	0.027	0.716

Units: dietary folate (μg/1000 Kcal/d), supplemental folic acid (Yes vs. No), total folate (μg/1000 kcal/d)

1 *P* for heterogeneity was determined in case-only analysis comparing mutated cases to nonmutated cases. The results are sorted from lowest to highest *P* value.

2 Benjamini–Hochberg correction applied to *P* for heterogeneity (*Q*-value).

No significant differential associations were observed for nonhypermutated tumors (Supplemental Tables 5–7). The associations with dietary folate were nonsignificant for all tested pathways in both hypermutated and nonhypermutated tumors (Table 3). Supplemental folic acid and similarly total folate showed null or inverse associations for the pathways. Overall, we did not observe any differential associations for the pathways tested. We also did not observe any significant associations of folate variables and CRC risk according to mutation burden of the tumors (hypermutated compared with nonhypermutated) (Supplemental Table 8).

**TABLE 3:** Associations between folate variables and colorectal cancer risk according to signaling pathways.

	Mutated cases vs. controls				Nonmutated cases vs. controls				*P* for heterogeneity^1^	BH corrected *P* value^2^
	Cases	Controls	OR (95% CI)	*P* value	Cases	Controls	OR (95% CI)	*P* value		
**Hypermutated tumors**										
Dietary folate										
IGF2/PI3K	318	9388	1.06 (0.89, 1.26)	0.533	312	9388	0.95 (0.80, 1.14)	0.591	0.901	0.993
MMR	262	9388	1.05 (0.87, 1.28)	0.617	368	9388	0.98 (0.83, 1.15)	0.760	0.931	0.993
RTK/RAS	475	9388	1.06 (0.92, 1.23)	0.402	155	9388	0.83 (0.65, 1.07)	0.143	0.165	0.880
TGF-β	526	9388	0.99 (0.86, 1.14)	0.910	104	9388	1.06 (0.79, 1.42)	0.701	0.957	0.993
WNT	611	9388	0.99 (0.87, 1.13)	0.866	19	9388	1.67 (0.86, 3.25)	0.131	0.085	0.878
TP53	304	9388	0.96 (0.81, 1.15)	0.691	326	9388	1.04 (0.88, 1.23)	0.655	0.917	0.993
Supplemental folic acid										
IGF2/PI3K	327	8970	0.86 (0.68, 1.10)	0.221	314	8970	0.70 (0.55, 0.89)	0.004	0.598	0.993
MMR	273	8970	0.86 (0.66, 1.12)	0.255	368	8970	0.72 (0.57, 0.91)	0.005	0.507	0.981
RTK/RAS	483	8970	0.80 (0.66, 0.98)	0.034	158	8970	0.70 (0.49, 0.98)	0.038	0.751	0.993
TGF-β	539	8970	0.77 (0.64, 0.94)	0.008	102	8970	0.80 (0.53, 1.21)	0.281	0.340	0.944
WNT	619	8970	0.78 (0.65, 0.94)	0.008	22	8970	0.58 (0.23, 1.47)	0.252	0.124	0.878
TP53	309	8970	0.70 (0.55, 0.90)	0.006	332	8970	0.85 (0.67, 1.08)	0.172	0.299	0.944
Total folate										
IGF2/PI3K	351	10,263	0.98 (0.84, 1.14)	0.781	351	10,263	0.77 (0.67, 0.90)	0.001	0.801	0.993
MMR	289	10,263	0.94 (0.80, 1.11)	0.493	413	10,263	0.82 (0.72, 0.95)	0.006	0.335	0.944
RTK/RAS	529	10,263	0.90 (0.79, 1.02)	0.084	173	10,263	0.78 (0.63, 0.97)	0.024	0.477	0.981
TGF-β	586	10,263	0.87 (0.77, 0.98)	0.023	116	10,263	0.86 (0.68, 1.11)	0.247	0.196	0.887
WNT	678	10,263	0.87 (0.78, 0.97)	0.011	24	10,263	0.98 (0.55, 1.74)	0.943	0.386	0.944
TP53	342	10,263	0.84 (0.72, 0.97)	0.021	360	10,263	0.90 (0.78, 1.05)	0.179	0.793	0.993
**Nonhypermutated tumors**										
Dietary folate										
IGF2/PI3K	504	9388	0.99 (0.86, 1.15)	0.892	2194	9388	1.01 (0.93, 1.09)	0.899	0.901	0.993
MMR	72	9388	0.81 (0.55, 1.20)	0.291	2626	9388	1.01 (0.94, 1.08)	0.844	0.931	0.993
RTK/RAS	1513	9388	1.02 (0.94, 1.12)	0.614	1185	9388	0.98 (0.88, 1.08)	0.616	0.165	0.880
TGF-β	586	9388	1.00 (0.88, 1.15)	0.957	2112	9388	1.00 (0.93, 1.08)	0.979	0.957	0.993
WNT	2114	9388	0.97 (0.90, 1.05)	0.469	584	9388	1.11 (0.97, 1.26)	0.127	0.085	0.878
TP53	1666	9388	1.02 (0.94, 1.11)	0.661	1032	9388	0.98 (0.88, 1.08)	0.633	0.917	0.993
Supplemental folic acid										
IGF2/PI3K	491	8970	0.83 (0.68, 1.02)	0.073	2243	8970	0.82 (0.74, 0.92)	<0.001	0.598	0.993
MMR	69	8970	0.87 (0.52, 1.46)	0.594	2665	8970	0.82 (0.74, 0.91)	<0.001	0.507	0.981
RTK/RAS	1495	8970	0.80 (0.71, 0.90)	<0.001	1239	8970	0.86 (0.75, 0.98)	0.025	0.751	0.993
TGF-β	581	8970	0.76 (0.63, 0.91)	0.003	2153	8970	0.84 (0.76, 0.94)	0.002	0.340	0.944
WNT	2096	8970	0.79 (0.71, 0.88)	<0.001	638	8970	0.93 (0.78, 1.12)	0.450	0.124	0.878
TP53	1682	8970	0.88 (0.78, 0.99)	0.033	1052	8970	0.75 (0.65, 0.86)	<0.001	0.299	0.944
Total folate										
IGF2/PI3K	577	10,263	0.88 (0.77, 1.00)	0.052	2607	10,263	0.92 (0.86, 0.99)	0.021	0.080	0.878
MMR	82	10,263	1.10 (0.79, 1.54)	0.577	3102	10,263	0.91 (0.86, 0.97)	0.004	0.335	0.944
RTK/RAS	1759	10,263	0.92 (0.85, 1.00)	0.042	1425	10,263	0.91 (0.83, 0.99)	0.027	0.274	0.939
TGF-β	686	10,263	0.86 (0.76, 0.97)	0.012	2498	10,263	0.93 (0.87, 1.00)	0.045	0.853	0.993
WNT	2476	10,263	0.90 (0.84, 0.97)	0.003	708	10,263	0.96 (0.86, 1.08)	0.492	0.134	0.880
TP53	1972	10,263	0.93 (0.87, 1.01)	0.075	1212	10,263	0.89 (0.81, 0.97)	0.011	0.541	0.993

Units: dietary folate (μg/1000 Kcal/d), supplemental folic acid (Yes vs. No), total folate (μg/1000 kcal/d)

1 *P* for heterogeneity was determined in case-only analysis comparing mutated cases to nonmutated cases. The results are sorted from lowest to highest *P* value.

2 Benjamini–Hochberg correction applied to *P* for heterogeneity (*Q*-value).

## Discussion

Using data from 2 large consortia, we observed inverse associations between supplementary folic acid and total folate and CRC risk. We did not observe differential associations according to mutation status in the 105 tested genes and 6 signaling pathways, after correcting for multiple testing. Nonetheless, we observed some indication of nominal significance with 1 or more folate variables being differentially associated with CRC according to somatic mutations in *AXIN2*, *B2M*, *BCOR*, *CHD1*, *DOCK3*, *FBLN2*, *MAP3K21*, *POLD1*, *RYR1*, *TET2*, *UTP20*, and *ZNF521.* The associations between folate and CRC were mostly inverse or toward the null, except for *DOCK3-*, *CHD1-*, and *ZNF521*-mutated tumors for which we observed positive associations.

The inverse associations observed between total folate intake and CRC risk have been reported in previous epidemiologic studies and summarized in meta-analyses [8,9]. Folate may mitigate genetic and epigenetic changes by preventing global DNA hypomethylation, and genome instability [29]. Zsigrai et al. [30] have demonstrated that the effect of folic acid supplementation influences the genetic and epigenetic of CRC cell lines, and more importantly differentially targets several genes, hence could contribute to differential associations by molecular subtypes of the tumors. This is supported by experimental data from animal and mechanistic studies showing the relationship between folate intake and DNA repair and mutation rates in colonic tissues [10]. Folate has been shown to modulate the expression of genes involved in colonic cell cycle and various signaling pathways in in vitro experimentation [31]. Similar findings were also reported by Schernhammer et al. [32], who showed that folate intake was inversely associated with the incidence of long-interspersed nucleotide element-1 (*LINE-1*) hypomethylated colorectal tumors, whereas *LINE-1*-hypermutated tumors showed null associations further stressing the role of folate deficiency in specific carcinogenic mechanisms. In addition, the association between folate and CRC has been reported in cell lines analysis to be differential in DNA-repair-associated genes [33]. Nevertheless, previous epidemiologic studies have not shown differential associations between folate intake and CRC risk by *BRAF*, or *KRAS* status in 3 United States cohort studies i.e., the Iowa Women’s Health Study, the Nurses’ Health Study and the Health Professional Follow-up Study [34,35]. Overall, previous experimental studies have suggested that the role of folate in CRC may be gene- or pathway-specific, but observational epidemiologic studies did not report on these findings, either by using specific genes or exploring the associations according to broad molecular subtypes.

The genes with marginal differential associations in our study were not previously specifically associated with folate metabolism. For example, *DOCK3* (previously known as modifier of cell adhesion or presenilin-binding protein), a member of the DOCK180 family of guanine nucleotide exchange factors have not been consistently reported as a significant gene in folate metabolism or colorectal carcinogenesis. Although *DOCK3* is expressed in colon tissues, it is mostly known for its role in cytoskeleton organization and cell-matrix modeling [36], and as such has been reportedly investigated as a stimulator of axonal growth [37] or in conditions such as attention-deficit hyperactivity disorder [38]. Nonetheless, *DOCK3* has been shown to be mutated in CRC tumors and might, according to a genetic principal component analysis, serve with a panel of other genes such as *CACNA1D*, *SERPINB4*, and *ZBED6* as a subtype of CRC [39]. In this study, *DOCK3* was mutated alone in our study, thus suggesting potential specificity of this gene to be further explored. Although it is unclear how folate differentially affects *DOCK3*-mutated tumors, our findings are intriguing and warrant further investigation to validate and explain the role of this gene in the folate–CRC association.

*ZNF521* is a 30-zinc finger transcription cofactor with regulatory functions in the regulation of hematopoietic, adipose tissue and mesenchymal stem cells [40]. This gene has been widely reported in experimental tissue and animal studies for its expression in the brain and implications in brain structure development, regulatory effect of the cerebellum development, and differentiation of striatal neurons [41]. *ZNF521* is expressed in some cancers such as myeloid leukemia, in which it is tagged as a potential therapeutic pathway because of its role in DNA transcription [42]. *ZNF521* expression has also been associated with the prognosis of pediatric neuroblastoma [43], gastric cancer, CRC [44], and ovarian cancer [40]. Furthermore, it has been linked to accelerated differentiation in cell lines such as erythroid cell [45] or brain cells [46], whereas in other tissues such as adipose tissue it was reported to repress differentiation of stem cells [47]. Thus, *ZNF521* may act usually as a promoter or occasionally as a suppressor of transcription and cancer risk depending on the tissue and the cellular context. There exists growing evidence on the regulation of *ZNF521* in several cancers, and the recent advances in folate–miRNA relationships [48] could possibly provide additional mechanistic explanation of our finding in future studies.

Another significant finding was the significant positive association observed between total folate with *CHD1* in hypermutated tumors, whereas a null association was found in nonhypermutated tumors. There is no clear explanation for such differential associations and there is a scarce amount of evidence supporting the role of folate specifically related to *CHD1’*s mutation. *CHD1* belongs to the family of nucleosome remodeling ATPases, whose role is to assemble, slide, and remove nucleosomes from the DNA [49]. *CHD1* possesses 3 domains: N- and C-terminals that act as chromodomain pairs and DNA-binding domain, respectively, and a central ATPase motor. The latter binds to the active epigenetic methylation of histone 3 lysine 4 and participates in DNA unwrapping and increases its accessibility and transcriptional elongation [50]. It is known that *CHD1* is particularly expressed in microsatellite instability (MSI)-high colorectal tumors [51]. Moreover, folate status has also been increasingly associated with *CHD1*-linked pathways in cancers such as adenomatous polyposis coli/wingless (APC/WNT) pathway [31]. Furthermore, *CHD1* expression loss was associated with DNA repair impairment and accumulation of DNA breaks and *CHD1* is essential for the reprogramming of somatic cells [51]. Taken together, our findings of a differential association with a chromatin-remodeling factor such as *CHD1* [52] calls for additional studies to further understand the link between folate’s impact at the gene level to histone modification, chromatin remodeling, and colorectal carcinogenesis.

The main strength of our study was its large sample size coupled with the uniquely large, carefully designed panel of genes sequenced. In addition, we took advantage of the rigorous approach to data harmonization conducted across studies and were able to include dietary and supplemental folic acid in our analysis. A major limitation of our study was that it included only participants of European ancestry; thus, our findings may not be extrapolated to other populations. Another limitation is that supplemental folic acid use was missing for approximately one-fourth (27.0%) of the participants. In addition, folate variables were collected at 1 point in time and may not take into consideration possible changes in the diet or supplement use over the years. Furthermore, some folate supplements may contain other B vitamins, which could enhance folate’s physiologic actions. Finally, we did not have detailed information on cancer treatment on all the participants. Patients with rectal cancer commonly undergo neoadjuvant treatment consisting of radiotherapy with or without chemotherapy, but most participants with rectal cancer in our sample would have been diagnosed before neoadjuvant treatment became the standard of practice, and thus, neoadjuvant treatments wouldn’t have impacted the mutation profile of the tumor.

In conclusion, in this large-scale analysis of folate intake in relation to somatic mutations in CRC, we found little evidence of differential CRC risk according to the mutational status of all the genes tested, after accounting for multiple testing. Nonetheless, we observed some nominally significant differential associations in 12 genes, including positive associations between folate intake and the risk of *CHD1*-, *DOCK3*-, and *ZNF521*-mutated colorectal tumors, which warrant replication in future studies. Overall, our findings demonstrated that the role of folate in CRC may be far more complex than hypothesized, and dietary and supplemental folic acid may impact CRC risk depending on specific genes.

## Author contributions

The authors’ contributions were as follows – UP, BvG, KKT, AIP: designed research; CQ, RSS: data curation; UP, AIP, SO, LH, AET, HB, SIB, DDB, PTC, YC, ATC, DAD, JCF, AJF, SG, PG, MG, ELG, SBG, MJG, TAH, MH, WYH, MAJH, JRH, MAJ, BML, VM, NM, CCN, JAN, MOS, WS, TU, CYU, SHZ, KKT, BvG, UP; conducted research; UP, AIP, SO, LH, AET, HB, SIB, DDB, PTC, YC, ATC, DAD, JCF, AJF, SG, PG, MG, ELG, SBG, MJG, TAH, MH, WYH, MAJH, JRH, MAJ, BML, VM, NM, CCN, JAN, MOS, WS, TU, CYU, SHZ, KKT, BvG: designed the methodology; UP, AIP, BvG, SH: provided essential reagents or provided essential materials; EKA, CQ: analyzed data or performed statistical analysis; EKA, CQ, BvG, UP, AT, AIP, RSS, SO, HB, KTT: wrote paper; EKA, UP, KTT, BvG: had primary responsibility for final content; and all authors: read and approved the final manuscript.

## Conflict of interest

The authors report no conflicts of interest.

## Funding

The work of KKT was funded by a Wereld Kanker Onderzoek Fonds (WKOF) and by World Cancer Research Fund International (WCRF; IIG_FULL_2020_022) grant. Funding information for the individual studies included are presented in Supplementary materials.

## Data availability

The datasets supporting the current study have not been deposited in a public repository because they are part of an international consortium but are available from the corresponding author upon request.
